# Anti-Ro-Positive Dermatomyositis Presenting as a Clinical Mimic of Guillain-Barré Syndrome: A Case Report

**DOI:** 10.7759/cureus.73154

**Published:** 2024-11-06

**Authors:** Awab Ismail, Nekhal M Moiddin, Mohammed A Mahmood, Asad Ullah Khan, Neha Nagaraj

**Affiliations:** 1 Internal Medicine, Queen Elizabeth Hospital Birmingham, Birmingham, GBR; 2 Acute Internal Medicine, Good Hope Hospital, Birmingham, GBR; 3 Cardiology, Good Hope Hospital, Birmingham, GBR; 4 Respiratory Medicine, Good Hope Hospital, Birmingham, GBR; 5 Acute Medicine, Queen Alexandra Hospital, Portsmouth Hospital University NHS Trust, Portsmouth, GBR

**Keywords:** anti-ro52, anti-ro/ssa, dermatomyositis, guillain-barré syndrome (gbs), idiopathic inflammatory myositis, inflammatory myositis

## Abstract

Dermatomyositis is a multi-system, connective tissue immune-mediated inflammatory condition characterised by myositis and distinct skin manifestations, with a higher prevalence in women. Symptoms typically appear in adulthood, though a juvenile form exists. Early signs may include Gottron’s papules and heliotrope rash, with proximal muscle weakness as the second most common initial symptom.

This report discusses a case of a 55-year-old woman whose presentation initially resembled acute demyelinating polyneuropathy (ADP), beginning with an eight-week history of dry cough and a five-day history of diarrhoea for four weeks followed by muscle weakness and no skin signs. The patient, who had delayed medical attention, presented with significant proximal muscle weakness and absent reflexes, resulting in a debilitating reduction in her baseline mobility. Initial investigations that were conducted included an elevated creatinine kinase (CK) level and reduced forced vital capacity (FVC). Her cerebrospinal fluid (CSF) analysis showed elevated proteins. She was admitted to the intensive care unit due to a declining FVC, and receiving intravenous immunoglobulin (IVIG), which resulted in some improvement. Over time, she developed skin manifestations and responded to treatment with mycophenolate mofetil (MMF) and corticosteroids.

## Introduction

Dermatomyositis is a rare condition that can occur alone or associated with underlying malignancy, and its diagnosis is based on clinical criteria, including skin findings and muscle weakness. 

It typically presents in early to middle adulthood, except for in the juvenile form. The diagnosis of dermatomyositis is made based upon meeting set clinical criteria. The Myositis Organisation describes a diagnostic criterion that includes at least one characteristic skin finding: heliotrope rash, shawl sign, periungual telangiectasia, and consistent skin biopsy findings. Alongside this, at least four of the following clinical features are required to be present: symmetrical muscle weakness, elevation of creatinine kinase (CK) or other skeletal muscle-associated enzyme, muscle pain, consistent electromyography (EMG) changes, presence of myositis antibodies on blood testing, arthralgia or non-destructive arthritis, signs of systemic inflammation, and consistent muscle biopsy findings [1]. There have been rare case reports of Guillain-Barré syndrome (GBS) occurring with an inflammatory myositis at the same time [2, 3]. Multiple myositis-specific antibodies have been implicated in the diagnosis of dermatomyositis, of which Anti-Ro52 has been shown to be associated with a more severe form of concurrent interstitial lung disease [4]. 

Acute inflammatory demyelinating polyneuropathy, commonly known as GBS, is an acute immune-mediated condition characterized by inflammatory demyelination of the peripheral nervous system [5]. Whilst the main presenting features of dermatomyositis also include symmetrical motor weakness, this is usually associated with preceding characteristic skin findings that aren't present in GBS [1,6].

The diagnosis is primarily clinical, requiring symmetrical, progressive motor weakness and absent deep tendon reflexes that typically worsen within four weeks of onset. Prompt recognition is essential, as delay in treatment has been associated with worse outcomes [7]. Other components to consider that solidify the diagnosis include the absence of an alternative diagnosis, nerve conduction studies (which are only suggestive or supportive after at least two weeks of symptoms), blood tests (anti-ganglioside antibody testing), and respiratory signs such as a reduction in forced vital capacity (FVC) [5]. 

The European League Against Rheumatism (EULAR), along with collaborators, created a diagnostic classification score for idiopathic inflammatory myopathies (IIMs) [8]. This is a useful tool for firstly clarifying the probability that a patient has IIM based on clinical signs and investigation results and has been accepted as a highly sensitive tool used internationally [8]. 

Treatment of IIM in the adult sub-types includes high-dose steroids at induction during acute active myositis and disease-modifying treatment to maintain remission of the condition. Medications used as disease-modifying treatments include methotrexate, azathioprine, mycophenolate mofetil (MMF), ciclosporin, and tacrolimus [9]. 

## Case presentation

This case report of a 55-year-old patient initially presented with an eight-week history of non-productive cough and a five-day period of loose stools occurring four weeks prior to presentation, followed by progressive limb weakness, ultimately leading to significant disability in her mobility due to severe muscle weakness and being unable to stand from a seated position. She denied any B symptoms. She also had no difficulty swallowing, difficulty keeping her head up, or difficulty speaking. She had no significant past medical history and took no regular medications. She was a vaper (previously 10 pack-year tobacco history). 

Clinical examination revealed marked symmetrical proximal motor weakness pronounced in hip flexion and shoulder abduction with global absent reflexes but no skin or sensory abnormalities. Initial differential diagnoses included GBS and inflammatory myositis. After receiving intravenous immunoglobulin (IVIG), she showed improvement, but further tests indicated myopathy. 

Initial investigations demonstrated that her CK was raised to 14,265 U/L (units per litre), which was put down to reduced mobility. Her initial bedside FVC was 2 litres; however, serial testing identified a reduction down to 1.45 litres without any changes to her oxygen saturation or work of breathing. 

Due to the decline in her FVC, she was briefly monitored in the intensive therapy unit (ITU) but did not require ventilator support. Presumed to be GBS, she was given a five-day course of IVIG. Her cerebrospinal fluid (CSF) results showed a protein level of 0.31 mg/ml (milligrams per millilitre), less than 1 polymorph per mm^3^, less than 1 lymphocyte per mm^3^, normal glucose level, and negative culture and viral polymerase chain reaction (PCR). Her viral serology blood tests (testing for Epstein-Barr virus (EBV), cytomegalovirus (CMV), HIV, and hepatitis serology) were all negative. She showed objective improvement in her symptoms as she was ambulating with physiotherapy after completion of her IVIG course. 

The initial working differential diagnoses were GBS or IIM. Further investigations were conducted, including a nerve conduction study, myositis antibody panel blood tests, MRI of the shoulder and thighs, and transfer to the nearest tertiary centre for a muscle biopsy. 

**Figure 1:**
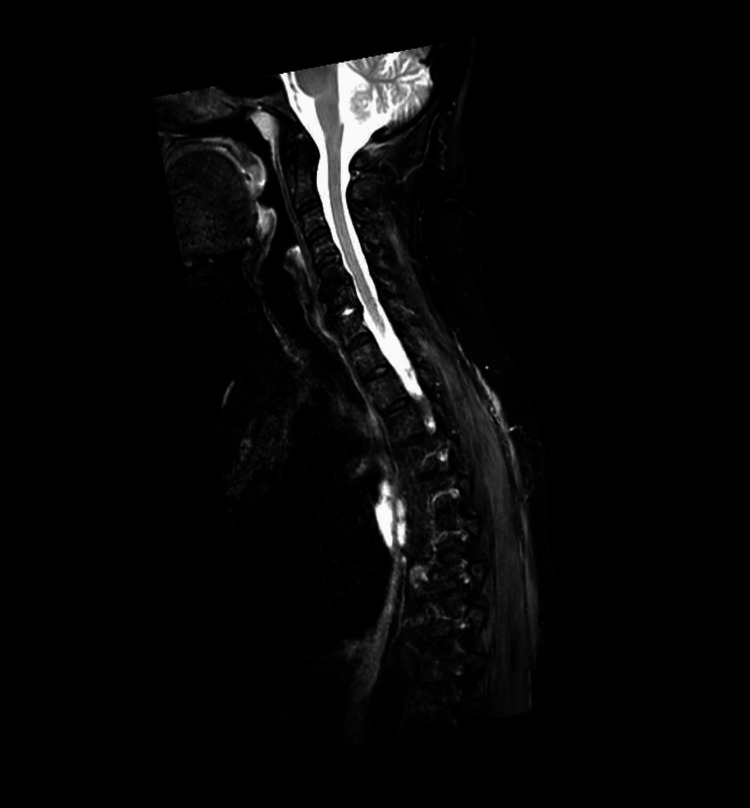
MRI of the cervico-thoracic spine (T2 sequence, sagittal view) showing para-spinal muscle oedema

**Figure 2:**
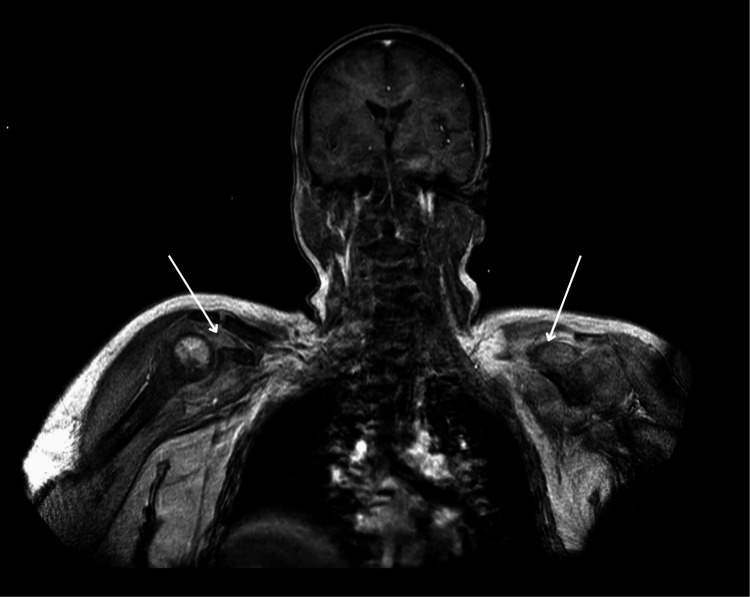
MRI of the upper thorax and shoulder region with contrast (T2 sequence, coronary view) showing hyper-intensity of the shoulder muscles surrounding the shoulder girdle, indicating active myositis.

**Figure 3:**
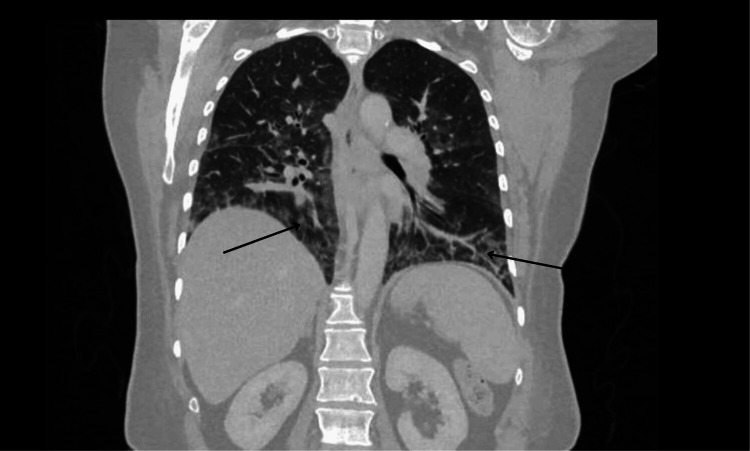
High-resolution CT of the thorax (coronal view) showing bilateral ground-glass changes, reticulations, and inter-lobular septal thickening. Findings are suggestive of interstitial lung disease.

An MRI of the head with contrast was unremarkable, whilst an MRI of the spine (T2 sequence) demonstrated paraspinal muscle oedema (Figure 1). An MRI of her shoulder T2 sequence supported these findings (Figure 2). She then underwent a muscle biopsy, which revealed myopathy with perimysium inflammation and scattered necrosis, a picture consistent with immune myopathy with perimysium pathology. Nerve conduction study results were also consistent with a diffuse myopathy picture. Auto-immune screen sent, including myositis-specific antibodies, returned negative, aside from the below-specified antibodies. A CT scan of the chest showed signs of non-specific interstitial disease (Figure 3). 

Her significant blood test results were extractable nuclear antibody (ENA) positive and Anti-Ro52 (associated with dermatomyositis with concurrent interstitial lung disease). Spirometry showed a restrictive pattern. Based on her blood test findings, spirometry results, and CT findings, all supported a diagnosis of dermatomyositis, or another inflammatory myositis, given the absence of skin findings at that time. 

At a two-month follow-up appointment with a neurologist, she was found to have a worsening cough and progressing limb weakness. She was then given intravenous methylprednisolone (IVMP) and referred to a rheumatologist, who later started her on MMF 500 mg twice daily, which was later increased to 1 g twice daily, along with a tapering dose of steroids. 

Of note, it was only during this second admission did the patient display cutaneous changes in the form of widespread inflammatory skin rash and nail fold vasculitis. She responded well to this treatment regimen with the resolution of the rash and improvement in her motor power. An outpatient pulmonary function test showed improvement in the FVC, and the patient was followed up routinely in a rheumatology clinic. 

## Discussion

The presented case initially displayed multiple features highly suggestive of GBS. Particularly, the presence of an antecedent event (diarrhoeal illness), the presence of symptoms spanning over four weeks duration, and the symmetrical progressive motor weakness, as well as the areflexia, made it the main differential on initial presentation. On review of the literature, similar presentations with no initial skin manifestations and areflexia are a rare occurrence. In one case report, there was an associated acute myositis with concurrent GBS [2]. However, in that specific case report, both the acute myositis and the concurrent GBS were linked to acute infection with CMV. In one other case report in 2023 [3], there was a similar presentation of a patient with anti-nuclear matrix protein 2 (NPX-2)-associated inflammatory myositis, which presented as progressive limb weakness and areflexia, initially managed as GBS. The presence of anti-Ro52 has been associated with dermatomyositis with concurrent interstitial lung disease [4]. The presence of an antecedent event within six weeks of motor weakness has been shown to display high specificity (89%-100%) and significant sensitivity (13%-18%) for GBS [5]. Furthermore, normal CSF protein can occur in the first week of GBS and does not exclude the diagnosis [5]. On review of diagnostic criteria [1], the patient did not fulfill the required criteria on initial presentation to make a confident diagnosis of dermatomyositis. 

Dermatomyositis usually presents initially with skin manifestations and commonly precedes muscle weakness. Muscle weakness may sometimes occur weeks or months after skin manifestations occur [1]. Anti-Ro52 is a myositis-associated antibody that is responsible for the regulation of interferon type 1 and has been implicated in more severe connective tissue diseases [6]. It has also been shown to be associated with interstitial lung disease in dermatomyositis [6]. All of these features became apparent with further investigation in this case presentation, and formal spirometry and early CT scan of the thorax may have directed us towards the correct diagnosis earlier on in the disease trajectory. 

Hyporeflexia and areflexia, such as in this case, an unusual feature of dermatomyositis and is only found in severe, long-standing cases [6]. In this patient, the atypical presentation with the objective decline in respiratory function initially, managing this case initially as GBS was justified, as delays in treatment of GBS have been associated with worse outcomes [7].

Based on the EULAR/American College of Rheumatology (ACR) classification score of IIMs [8], on initial presentation in the absence of clinical signs, taking into account the muscle biopsy findings, this patient has a 67% probability of having IIM, and the likely subgroup is polymyositis.

## Conclusions

Dermatomyositis is a rare connective tissue disease that commonly presents with characteristic skin signs and symmetrical muscle weakness. In patients presenting with symmetric progressive weakness and a significantly elevated CK, immune-mediated inflammatory myositis should always be investigated and considered. When the diagnosis of presumed GBS is uncertain, nerve conduction studies and CSF analysis can both be vital tools in differentiating GBS from other conditions but should not delay treatment in someone who fits the clinical criteria and has an acute decline in respiratory function. Early diagnosis and commencement of treatment have been associated with improved outcomes in GBS.
